# Conceptual and Numerical Modeling of Radionuclide Transport and Retention in Near-Surface Systems

**DOI:** 10.1007/s13280-013-0399-1

**Published:** 2013-04-26

**Authors:** Àngels Piqué, David Arcos, Fidel Grandia, Jorge Molinero, Lara Duro, Sten Berglund

**Affiliations:** 1 CAT ENVIRO Geochemical Consultancy, Pl. Catalunya 4, 08810 Sant Pere de Ribes, Barcelona Spain; 2 Amphos 21, Passeig de Garcia i Faria 49-51, 08019 Barcelona, Spain; 3 HydroResearch AB, Stora Marknadsvägen 15S, 12th Floor, 183 34 Täby, Sweden

**Keywords:** Radionuclide, Safety assessment, Quaternary sediments, Reactive transport modeling, Solid/liquid distribution coefficient

## Abstract

Scenarios of barrier failure and radionuclide release to the near-surface environment are important to consider within performance and safety assessments of repositories for nuclear waste. A geological repository for spent nuclear fuel is planned at Forsmark, Sweden. Conceptual and numerical reactive transport models were developed in order to assess the retention capacity of the Quaternary till and clay deposits for selected radionuclides, in the event of an activity release from the repository. The elements considered were carbon (C), chlorine (Cl), cesium (Cs), iodine (I), molybdenum (Mo), niobium (Nb), nickel (Ni), radium (Ra), selenium (Se), strontium (Sr), technetium (Tc), thorium (Th), and uranium (U). According to the numerical predictions, the repository-derived nuclides that would be most significantly retained are Th, Ni, and Cs, mainly through sorption onto clays, followed by U, C, Sr, and Ra, trapped by sorption and/or incorporation into mineral phases.

## Introduction

Geological repositories for high level nuclear wastes are designed to contain radionuclides and, in the event of a release, retard their migration to the surface for very long time periods. The Swedish Nuclear Fuel and Waste Management Company (SKB) has selected a site in central Sweden, Forsmark, to host the geological repository planned for spent fuel from the Swedish nuclear power plants. The storage concept is based on deep disposal (at ~500 m) in a granitic bedrock environment. As part of the license application for the spent fuel repository, SKB performed an assessment of long-term radiological safety (Kautsky et al. 2013). Quantification of radionuclide transport and associated doses to man and the environment was an important part of the assessment.

Scenarios of barrier failure and radionuclide release to the near-surface environment are important to consider within performance and safety assessment exercises. In these and other applications involving contaminant transport by groundwater, solid/liquid distribution coefficients (*K*_d_) are widely used to describe the retention of contaminants, i.e., sorption and other processes retarding their migration (Sohlenius et al. 2013). The application of *K*_d_-based models relies on assumptions of linearity and equilibrium of the processes involved. It has been argued that this type of model can be considered reliable if the groundwater system is in a steady state of chemical evolution (Reardon 1981). However, in the case of radionuclide release and transport from a deep geological repository, transport conditions may not be consistent with the assumptions underlying the application of *K*_d_-based models (chemical disequilibrium will occur), and reactive transport models could be needed to complement or replace them.

Reactive transport models are powerful tools that couple groundwater flow, transport of solutes, and geochemical reactions between solid and aqueous phases. These numerical models can provide reliable quantitative evaluations for performance and safety assessment of a deep geological repository. The main objective of this study is to assess the capacity of the Quaternary deposits to retain carbon (C), chlorine (Cl), cesium (Cs), iodine (I), molybdenum (Mo), niobium (Nb), nickel (Ni), radium (Ra), selenium (Se), strontium (Sr), technetium (Tc), thorium (Th), and uranium (U), by means of conceptual and numerical reactive transport models. The paper presents site-specific process identification and conceptual modeling based on evaluation of an extensive dataset from the Forsmark site, followed by reactive transport simulations integrating retention processes with advective–dispersive transport by groundwater.

## Materials and Methods

The radionuclides studied here were selected based on relevance for the SKB safety assessment, and for representing different types of geochemical behavior. Although some short-lived nuclides, such as ^90^Sr, were considered, radioactive decay was not implemented in the numerical simulations. This also means that radionuclide sources in the form of decay chains were neglected.

The development of a conceptual model of radionuclide behavior in the Quaternary deposits requires the evaluation of the most feasible retention mechanisms, taking into account the available geochemical and hydrochemical data from the site and complementary data from similar environments. Once the conceptual model is built up, the selected retention processes for each element can be implemented in numerical simulations, in order to investigate their potential effects on radionuclide migration.

### Site Overview

The transfer of radionuclides from the bedrock to surface systems may take place in different geological environments at Forsmark (Hedenström and Sohlenius 2008): (i) direct contact between the crystalline bedrocks and biosphere–atmosphere (13 % of areal extent), (ii) carbonate-rich till, which is the most abundant Quaternary deposit (~65 % of surface extent), and (iii) Quaternary lake and wetland sediments. Lake sediments commonly overlie glacial till. The thickness of Quaternary deposits is relatively small but highly variable, usually from 0 to 2–3 m, although in some localities exceeding 10 m.

Glacial till is basically a mixture of calcite (20–30 wt%), quartz and clay minerals (mainly illite). In addition, sediment analysis suggests the presence of minor amounts of other minerals, such as Fe(III) oxyhydroxides (Tröjbom and Söderbäck 2006). The lake sediments consist of anoxic glacial clays and biogenic-derived sediments (gyttja) with abundant organic matter. The extensive microbial activity expected in this environment can eventually lead to the precipitation of iron sulfides, as observed in similar environments (Belzile et al. 2000).

The groundwater flowing through the till has high contents of calcium and bicarbonate (ionic strength, *I* ~ 1.0 × 10^−2^ mol L^−1^) and neutral pH, reflecting equilibrium with the solid material. Glacial clay porewater is believed to be more diluted (*I* ~ 6.0 × 10^−3^ mol L^−1^), with a high content of organic compounds, either dissolved or as suspended particulate ([DOC] ~ 1.5 × 10^−3^ mol L^−1^). Deep groundwaters, which are expected to be the potential radionuclide carrier in case of repository failure, are of Na–Cl type and much more saline.

### Brief Description of Numerical Models

**Fig. 1:**
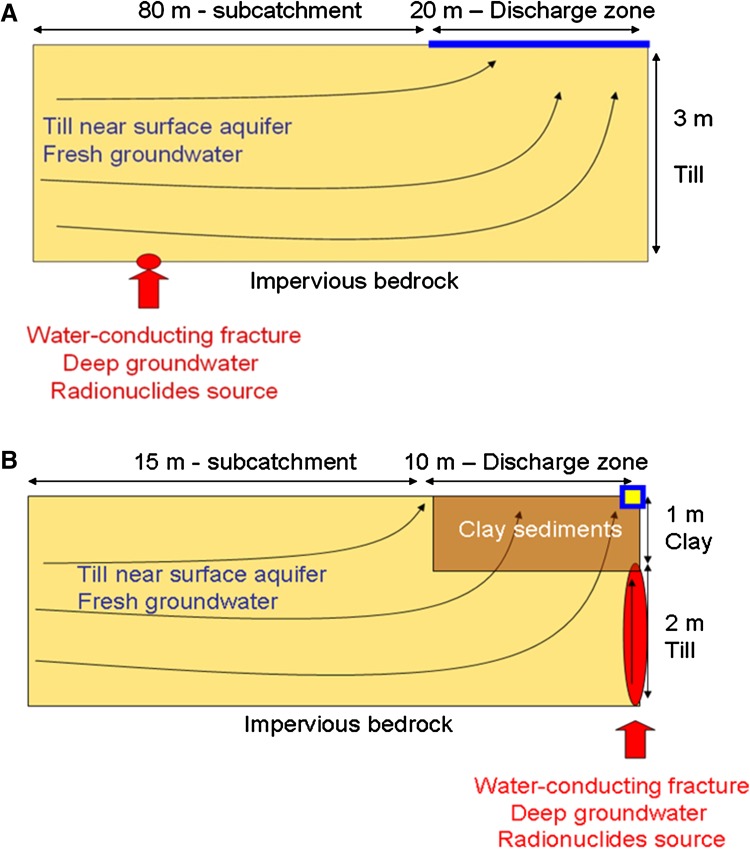
**A** Outline of the two-dimensional till domain considered in the numerical modeling. Computed breakthrough curves were evaluated over the whole discharge area of the domain. **B** Outline of the two-dimensional clay domain. Computed breakthrough curves were evaluated at the *blue* and *yellow* point

The retention capacity of the Forsmark Quaternary deposits was evaluated by means of reactive transport simulations, considering two distinct domains: (i) a till deposit overlying the granite bedrock (Fig. 1a), and (ii) glacial clay overlying till in a discharge zone, e.g., an area with a lake and/or wetland (Fig. 1b). The radionuclide-bearing groundwater was assumed to flow upwards through a fracture in the bedrock to reach the Quaternary deposits. In the modeling of the clay domain, transport through the underlying till was neglected, in order to focus on the retention processes in the clay. Hydrodynamic processes and parameters were based on MIKE SHE model results (Graham and Butts 2005; Butts and Graham 2008; Berglund et al. 2013).

The major reactive minerals initially present in both model domains were calcite and illite. Calcite contained trace amounts of Sr, forming a solid solution. The amount of illite was set to 10 wt% in the till and to 50 wt% in the clay domain, and only participated as a charged surface for sorption. Ferrihydrite was considered the redox controlling phase of the till porewater, with an assumed initial concentration of 0.1 wt%. For the clay domain, an initial concentration of 1.5 wt% of pyrite was used, in order to ensure reducing conditions. The initial composition of till and clay porewaters and the composition of the deep groundwater before and after the radionuclide release from repository are presented in Table 1. In the clay system, dissolved humic acids were included to simulate U and Th complexation in waters with high concentrations of dissolved organic compounds. Transport of colloids, either organic or inorganic, is out of the scope of this paper.

**Table 1:** Initial composition of porewater in the till and clay domains, and composition of deep groundwater before and after repository failure. RD denotes repository-derived radionuclides. Concentrations given in mol L^−1^

Parameter	Till porewater	Clay porewater	Deep GW	Deep GW (after repository failure)
pH	7.12	7.75	6.86	6.86
pe	0.053	−3.94	−2.58	−2.58
Na	1.22 × 10^−3^	2.64 × 10^−4^	6.13 × 10^−2^	6.13 × 10^−2^
K	1.22 × 10^−4^	5.19 × 10^−5^	8.00 × 10^−4^	8.00 × 10^−4^
Ca	2.79 × 10^−3^	1.18 × 10^−3^	1.82 × 10^−2^	1.82 × 10^−2^
Mg	3.54 × 10^−4^	1.17 × 10^−4^	4.73 × 10^−3^	4.73 × 10^−3^
Sr	2.10 × 10^−6^	6.23 × 10^−7^	5.24 × 10^−5^	5.24 × 10^−5^
Ba	7.28 × 10^−7^	1.37 × 10^−7^	4.38 × 10^−7^	4.38 × 10^−7^
C(IV)	5.58 × 10^−3^	2.50 × 10^−3^	4.72 × 10^−3^	4.72 × 10^−3^
Cl	1.90 × 10^−3^	1.53 × 10^−4^	1.04 × 10^−1^	1.04 × 10^−1^
SO_4_^2−^	2.41 × 10^−4^	6.39 × 10^−5^	2.21 × 10^−3^	2.21 × 10^−3^
Fe	1.75 × 10^−5^	8.35 × 10^−7^	5.80 × 10^−5^	5.80 × 10^−5^
NH_4_^+^	6.62 × 10^−6^	1.82 × 10^−5^	7.28 × 10^−5^	7.28 × 10^−5^
U	2.23 × 10^−8^	1.12 × 10^−9^	1.13 × 10^−8^	5.66 × 10^−9^
Cs	6.48 × 10^−11^	4.51 × 10^−11^	3.65 × 10^−9^	3.65 × 10^−9^
I	6.40 × 10^−8^	5.28 × 10^−5^	3.36 × 10^−7^	3.36 × 10^−7^
Nb	1.14 × 10^−9^	8.72 × 10^−11^	–	–
Ni	4.42 × 10^−8^	4.89 × 10^−9^	7.17 × 10^−9^	7.17 × 10^−9^
Th	6.09 × 10^−10^	1.51 × 10^−10^	1.19 × 10^−9^	1.19 × 10^−9^
Humic acid	–	1.00 × 10^−4^	–	–
^RD^Cs	–	–	–	3.48 × 10^−7^
^RD^U	–	–	–	5.66 × 10^−9^
^RD^Sr	–	–	–	1.56 × 10^−3^
^RD^I	–	–	–	1.58 × 10^−5^
^RD^Nb	–	–	–	5.25 × 10^−8^
^RD^Ni	–	–	–	4.96 × 10^−7^
^RD^Th	–	–	–	1.75 × 10^−7^
^RD^Cl	–	–	–	5.05 × 10^−7^
^RD^C	–	–	–	2.89 × 10^−7^
^RD^Ra	–	–	–	9.15 × 10^−11^
^RD^Se	–	–	–	3.77 × 10^−11^
^RD^Tc	–	–	–	5.27 × 10^−9^

The reactive transport simulations were performed with the code PHAST, version 1.5.1 (Parkhurst et al. 2004), and using the thermodynamic database reported in Duro et al. (2006) with revised data for selected species (see details in Piqué et al. 2010). A 2700-year period was first simulated, in order to reproduce the present-day conditions observed at the near-surface systems at Forsmark. After this period, the release of radionuclides was modeled by a continuous injection of deep groundwater, for a period of 30 000 years. Such a long time was used for determining effective *K*_d_ values, although the detailed analysis of computed results was done only for the first 2700 years.

Due to the geochemical variability of the Quaternary deposits, and in order to represent relevant future conditions, a sensitivity analysis was developed for important geochemical parameters: concentration of (Ca,Sr)CO_3_ solid solution, of illite sorption sites and of dissolved humic acid. Temperature variations were also considered, which could affect radiocarbon retention. For details on the modeling assumptions, parameters, hydrological and hydrogeochemical initial conditions, calculation of the initial concentrations of repository-derived radionuclides, and spatial and temporal discretizations, the reader is referred to Piqué et al. (2010) and Grandia et al. (2011).

### Quantification of Retention

The efficiency of the domain for radionuclide retention (*E*) can be quantitatively evaluated with the following equation: *E* = 100(1 − *C* ^R^/*C* ^C^), where *C* ^R^ is the concentration of a solute at a given node and time in the reactive transport simulation, and *C* ^C^ is the concentration of the same solute at the same node and time in a simulation without retention processes (referred to as the ‘conservative’ simulation below). E was calculated at the discharge area of each domain (see Fig. 1 for location).

The retardation factor (*R*) can be calculated as: *R* = (*T*_1/2_^R^)·(*T*_1/2_^C^)^−1^, where *T*_1/2_^R^ is the advective travel time of the solute in the reactive transport simulation, and *T*_1/2_^C^ is the advective travel time of the same solute in the conservative simulation (without retention processes). (Advective travel times are derived from breakthrough curves at the discharge area of each domain.) The retardation factor can be interpreted as the ratio of transport velocities of reactive and conservative solutes; as shown below it can also be related to *K*_d_.

The *K*_d_ value is defined as the ratio between the element concentrations (*C*) in the solid and liquid phases: *K*_d_ = *C*_solid_/*C*_solution_, in L kg^−1^. The *K*_d_ model used here considers that the retention of an element in the solid phase includes sorption and precipitation (either as pure phase or solid solution), which are assumed to be reversible processes in the time frame of the calculations.

Effective *K*_d_ (denoted *K*_de_) can be calculated using retardation factors obtained from breakthrough curves: *K*_de_ = (*R* − 1)·(*φ*_e_)·(*ρ*_b_)^−1^, where *φ*_e_ is the effective porosity and *ρ*_b_ is the dry bulk density of the material. The effective porosity used here was 0.079 for the till and 0.2 for the clay system. The dry bulk density was 1.95 kg L^−1^ for the till and 2.0 kg L^−1^ for the clay system (see Piqué et al. 2010 and references therein for a description and justification of porosity and density data selection).

## Development of a Conceptual Model for Radionuclide Retention in Forsmark

### Geochemical Behavior of Radionuclides in the Quaternary Deposits

This section summarizes the study of geochemical behavior of radionuclides in the Forsmark near-surface system and the thorough evaluation of their potential retention mechanisms in the Quaternary deposits, which are fully reported in Piqué et al. (2010).

Precipitation of carbonate minerals may be an effective sink for repository-derived C, Sr, Se, and Ni in the till layers (e.g., Lamble et al. 1995; Tesoriero and Pankow 1996; Lakshtanov and Stipp 2007). The strong positive correlation between Sr and Ca in the Forsmark till and shallow groundwater points to the occurrence of (Ca,Sr)CO_3_ solid solutions with a relatively constant Sr/Ca ratio, which are considered common solubility limiting phases for Sr^2+^ (Bruno et al. 2002).

According to Schmidtz and Aumann (1995) and Ashworth and Shaw (2006), organic matter can play a significant role in the retention of chloride and iodide (expected major species of Cl and I, respectively, in Forsmark near-surface groundwater). In agreement with this conclusion, the measured *K*_d_ values of Cl in Forsmark soils and lake sediments show a positive correlation with organic matter (Engdahl et al. 2008; Sheppard et al. 2009). Organic matter can form complexes with other repository-derived radionuclides, as they do with the natural isotopes, such as Th, Ni, U(VI), and Se (Langmuir and Herman 1980; Belzile et al. 2000; Reiller 2005; Zhou et al. 2005).

In the Forsmark till deposits, the slightly oxidizing conditions and near-neutral pH of groundwater favor the precipitation of ferric oxy-hydroxides, which can sorb Ni, U(VI), Th, Sr, and Se species (Payne et al. 1996; Trivedi and Axe 1999; Green-Pedersen and Pind 2000; Reiller et al. 2005; Duc et al. 2006). The clay minerals in the Quaternary deposits (mainly illite) can sorb several repository-derived radionuclides, such as isotopes of Th, Ni, Sr, U, Cs, and Ra (Sawhney 1972; Turner et al. 1996; Lu and Mason 2001; Shahwan and Erten 2004; Bradbury and Baeyens 2009a, b). Sorption onto illite is considered the major retention process for Cs^+^ (e.g., Poinssot et al. 1999).

Some elements are highly mobile in their oxidized forms, such as Mo as molybdate and Tc as pertechnetate. If anoxic conditions prevail, e.g., in Forsmark wetlands and lake sediments, the solubility of Tc can be dramatically decreased by the reduction of Tc(VII) to Tc(IV) and precipitation of Tc(IV) species (e.g., Burke et al. 2005). In anoxic environments, molybdate can be converted to thiomolybdate and be scavenged by iron-rich particles, sulfur-rich organic matter and iron sulfide (Tribovillard et al. 2004; Vorlicek et al. 2004).

Under reducing conditions, Th, Se, and U(IV) can be retained by precipitation of mineral phases. If enough Th is available in the system (in the order of 10^−8^ M), the precipitation of amorphous ThO_2_ can take place under near-neutral to basic pH. U(IV) solid phases are mainly oxides, especially uraninite. Se oxyanions can be reduced and precipitated as native Se, ferroselite, or Fe_1.04_Se (Bruggeman 2008; Scheinost et al. 2008). Se^2−^ and Ni^2+^ can replace S^2−^ and Fe^2+^, respectively, in newly formed sulfides.

Niobium seems to be strongly retained in Forsmark Quaternary deposits, based on *K*_d_ data (Engdahl et al. 2008; Sheppard et al. 2011). However, the processes responsible for this retention cannot be ascertained so far. Niobium could precipitate on silt particles (Charles and Prime 1983) or associate with iron oxides and/or organic matter (Åström et al. 2008). A correlation between dissolved Nb and Fe is observed in Forsmark.

Till groundwater in Forsmark is very close to barite saturation and the very low concentrations of aqueous Ra (from 10^−15^ to 10^−13^ M) indicate that its solubility is controlled by (Ba,Ra)SO_4_ solid solutions (Grandia et al. 2008). Therefore, the precipitation of this mineral can be a sink for repository-derived Ra^2+^.

### Retention Processes Implemented in the Numerical Models

Based on the conceptual evaluation of element retention in the Forsmark near-surface environment, processes identified as most likely to significantly affect radionuclide retention are presented in Table 2. The strength of the different processes likely to retain radioelements can be quantified through association, sorption, or equilibrium constants. Sorption onto organic matter was not simulated due to scarcity of thermodynamic data and the large uncertainty in the related parameters required for modeling. Molybdenum was not implemented in the modeling due to the lack of related data.

**Table 2:**
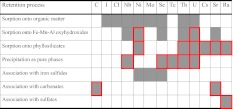
Retention processes that may be relevant for the selected elements in the Forsmark Quaternary glacial till and/or clay sediments (gray-shadowed). The processes implemented in the numerical modeling are marked in red

The retention processes included in the numerical modeling are also summarized in Table 2. In the till domain, sorption was assumed to take place onto illite and ferrihydrite. In the glacial clay domain, only sorption onto illite was considered. In both domains, the phases allowed to precipitate if saturation was reached were: (Ca,Sr)CO_3_, Nb_2_O_5_, native Se, ferroselite, Fe_1.04_Se, TcO_2_·1.6H_2_O, UO_2_·2H_2_O, schoepite, soddyite, uranophane, becquerelite, and radiobarite. Iodide and chloride were considered as conservative in the numerical modeling.

## Results and Discussion

### Quantitative Modeling of Radionuclide Migration

#### Breakthrough Curves and Radionuclide Retardation

**Fig. 2:**
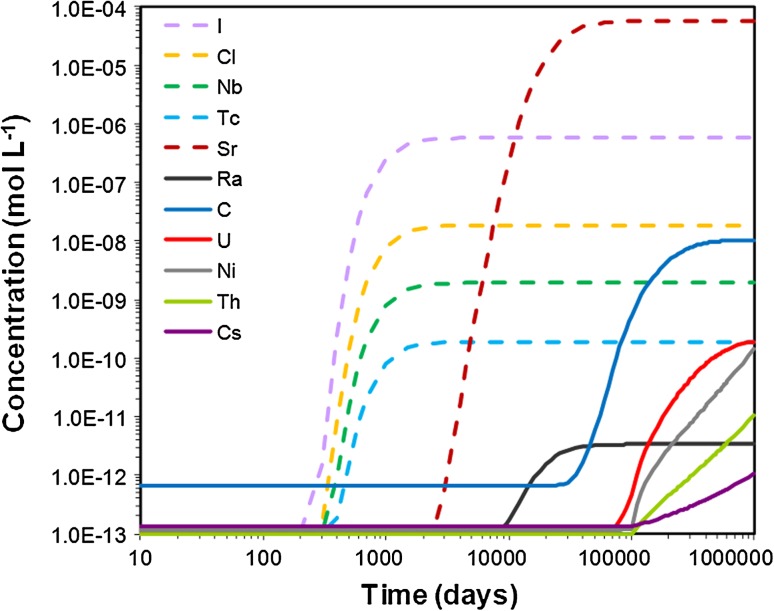
Integrated breakthrough curves of repository-derived elements at the discharge area of the till domain (see Fig. 1a for location)

**Fig. 3:**
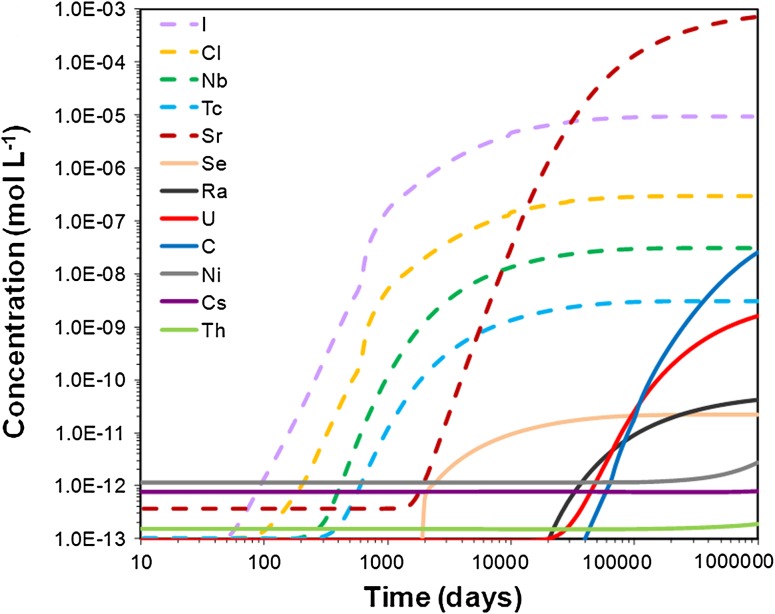
Breakthrough curves of repository-derived element concentrations at the observation point of the clay domain (see location in Fig. 1b)

The repository-derived radionuclides that behave conservatively (e.g., I and Cl) are expected to discharge very quickly to the surface water both in the till (Fig. 2) and clay system (Fig. 3). For these species, the steady state concentrations are reached approximately 500 years later in the clay system than in the till, due to the contrasting hydrogeological properties of the media (advection and dispersion are the dominant transport processes in the till system, whereas in the clay system diffusion plays a more important role).

Tc behaves conservatively in the modeled domains. In the till system, this is consistent with the fact that under oxidizing conditions the main Tc(VII) aqueous species is negatively charged (TcO_4_^−^). In the clay system, the more reducing environment favors the stability of Tc(IV) species, but the concentration of Tc in solution is too low for the precipitation of TcO_2_·1.6H_2_O.

Nb also behaves conservatively in the till and clay systems; under the considered Forsmark conditions, Nb_2_O_5_ solid phase is far from saturation. In the real case, iron oxides, organic matter and/or silt particles could retain Nb, although the retention mechanisms are not well known and could not be simulated.

For Se, the simulation shows that pure phases will not precipitate in the whole clay domain. However, the precipitation of pure phases is not the only mechanism that potentially can retain Se; the concentration of dissolved Se in the clay system could decrease, for example, by incorporation to newly formed sulfides. In the till domain, the Eh–pH conditions and the concentration of dissolved Se favor the precipitation of native Se, and the release of this radionuclide to the discharge area is prevented. It should be emphasized that the stability field of native Se under the conditions of interest is very narrow, and a slight change in Eh and/or pH would prevent its precipitation.

The next repository-derived elements discharging in both domains are Sr and Ra, with Ra arriving somewhat later in both systems. C and U follow, with U being delayed relative to C in the till system, while Cs, Th, and Ni show the strongest retardation (Figs. 2, 3).

The till system is not efficient in retaining Sr, which is only partially exchanged onto illite. Close to the deep groundwater discharge point, Sr is also incorporated into calcite. The clay system shows a decreasing capacity to retain Sr with time; after 2700 years, the maximum Sr concentration at the discharge area is only 25 % lower than in the corresponding simulation without retention processes (not shown). The sensitivity analysis shows that the timing of arrival of Sr at the discharge area is controlled by the amount of illite present in the sediment.

Radium is partially retained by cation exchange in both the till and clay sediments, and is also scavenged from solution by radiobarite precipitation in the till. The retention efficiency of Ra in the till drops very fast to almost zero, whereas such a decrease is more gradual in the clay domain, with a reduction down to 20 % after almost 3000 years of simulation. It is worth noting that slight changes in groundwater composition could preclude the precipitation of radiobarite and Ra uptake in the till.

For radiocarbon, the main retention mechanism considered is carbonate precipitation. The models predict that ^14^C could be efficiently retained in the clay system; after 2700 years of repository release, the dissolved ^14^C concentration at the discharge area is one order of magnitude lower in the reactive transport simulation than in the conservative simulation. On the contrary, the retention efficiency drops very fast in the till, and radiocarbon behaves conservatively during most of the simulation time. The sensitivity analysis at 5, 15, and 25 °C shows that isotopic fractionation processes do not change radiocarbon retention over this temperature range.

Uranium is another repository-derived radionuclide that is only partially retained in the simulated domains. For the till, the retention efficiency will be at its maximum in the beginning of the period of repository release and will significantly decrease with time (Table 3). Sensitivity analyses showed that a smaller number of illite sorption sites did not affect the concentration of U in solution, since it is mostly retained by ferrihydrite. In the clay domain, U is retained by precipitation of amorphous uraninite and sorption onto illite; the sensitivity analysis showed that a one order of magnitude increase in dissolved humic acids does not significantly change the concentration of U in solution.

**Table 3:** Retention efficiency (E), retardation factor (R), and effective *K*_d_ (*K*_de_; in L kg^−1^) of repository-derived elements at the discharge area of the simulated till and clay domains

Element	*E* (2700 years)		*R*		*K*_de_	
	Till (%)	Clay (%)	Till	Clay	Till	Clay
C	0.8	85	196	291	7.9	29
Cl	0	0	1	1	0	0
Cs	100	100	–	–	–	–
I	0	0	1	1	0	0
Nb	0	0	1	1	0	0
Ni	99	100	6035	–	244	–
Sr	0.02	24	24	30	0.9	2.9
Th	100	100	11 819	–	478	–
U	7.6	57	402	>158	16	>16
Ra	3.0	21	20	27	0.8	2.6
Se	100	0	–	1	–	0
Tc	0	0	1	1	0	0

**Fig. 4:**
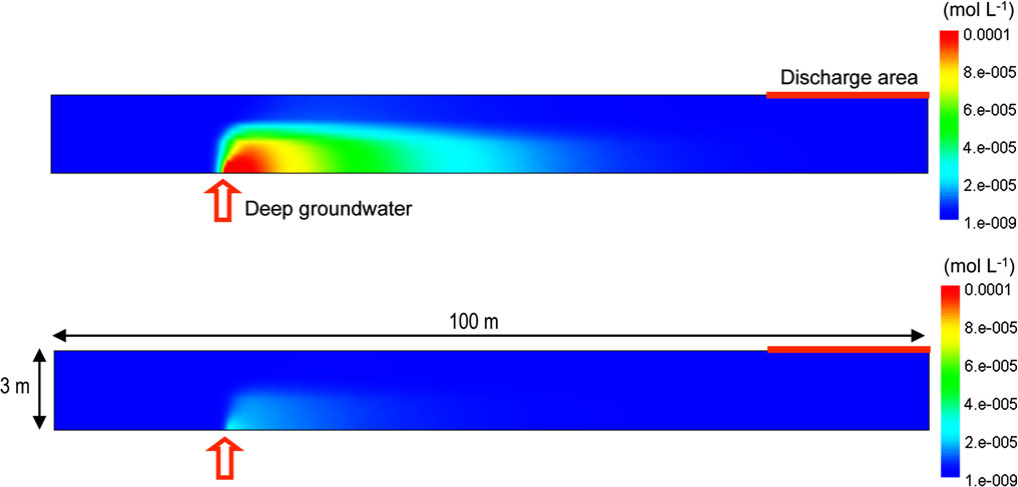
Repository-derived Ni^2+^ retained on illite (*upper panel*) and on ferrihydrite strong sorption sites (*lower panel*), after 2700 years of radionuclide release into the till domain

The most strongly retarded repository-derived elements in the modeled domains are Cs, Ni, and Th (computed retention efficiency of, or very close to, 100 % after 2700 years in both domains). These elements are very efficiently retained by illite, and Ni is also retained by ferrihydrite in the till domain (Fig. 4). The sensitivity analysis showed that a one order of magnitude decrease in the number of illite sorption sites produced a one order of magnitude increase of Th and Cs concentrations in solution and a two orders of magnitude increase for Ni in the clay system, and two orders of magnitude increase for Th and Cs and one order for Ni in the till system.

The retardation factor (*R*) is a parameter that indicates to what extent the radionuclides are retained relative to non-reactive solutes. Calculated *R* values are reported in Table 3. In some cases, the delay was so large that R could not be computed in the time frame of the model. This was the case for repository-derived Cs, Ni, and Th in the clay domain and for Cs and Se in the till. The sensitivity analyses revealed that retardation is correlated with the amount of sorption sites for those radionuclides that are mainly retained on illite; when the number of illite sorption sites is reduced by one order of magnitude, R decreases by up to one order of magnitude for Th and Ra. In contrast, the reduction of R is not so significant for U and Sr in the till, because other processes are also involved in their retention (sorption onto ferrihydrite and precipitation of (Ca,Sr)CO_3_, respectively).

#### Solid/Liquid Distribution Coefficients (*K*_d_)

**Fig. 5:**
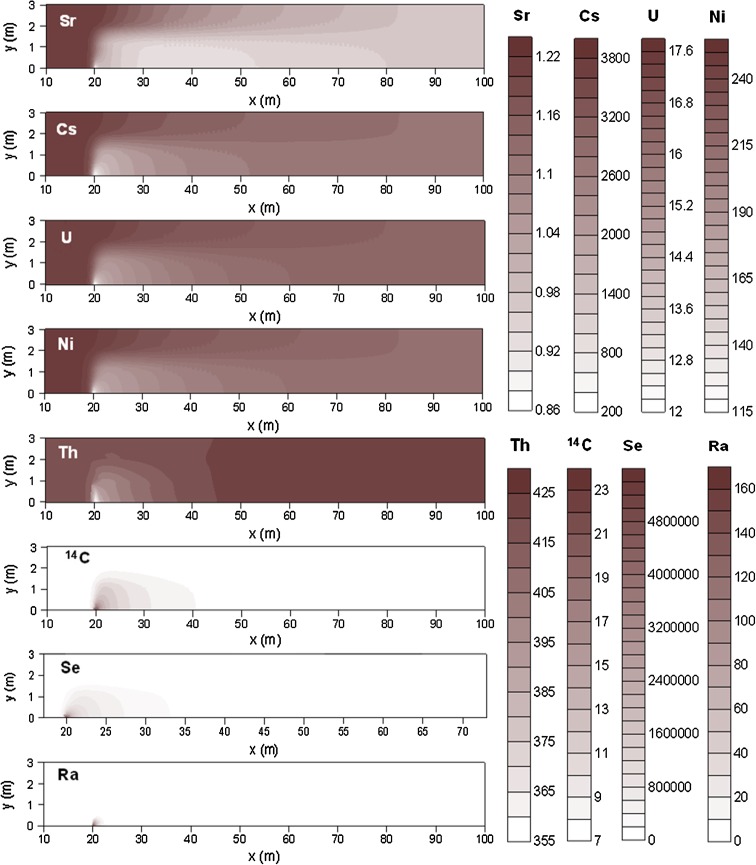
Distribution maps of *K*_d_ (L kg^−1^) for selected elements in the till domain after 2700 years of radionuclide release (see Fig. 1a for description of model domain)

**Fig. 6:**
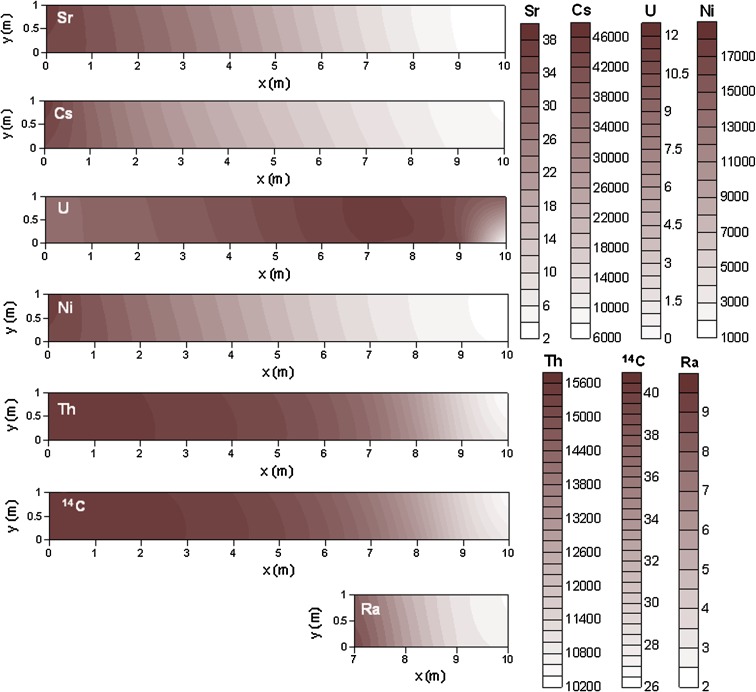
Distribution maps of *K*_d_ (L kg^−1^) for selected elements in the clay domain after 2700 years of radionuclide release (see Fig. 1b for description of model domain)

The calculated effective *K*_d_ values at the monitoring points of the discharge area are reported in Table 3. In order to investigate spatial heterogeneity in retention conditions, *K*_d_ values were calculated at each node of the model domains for each element of interest. The resulting ‘*K*_d_ maps’ of the till and clay domains are shown in Figs. 5 and 6, respectively. The computed *K*_d_ value of each natural isotope is equal to that of the corresponding repository-derived radionuclide. Carbon is the only exception since a slight, but not significant, difference in *K*_d_ is observed due to isotope fractionation processes.

Once the geochemical quasi-steady state has been reached, the *K*_d_ values of the selected elements are relatively stable in time, but not in space. In the till domain, the elements that are most strongly retained by sorption onto clay and ferrihydrite (Sr, Cs, U, Ni, Th) show a *K*_d_ decrease in the whole area affected by the deep groundwater (Fig. 5). In the cases of Se, ^14^C, and Ra, the maximum *K*_d_ values are reached in the vicinity of the deep groundwater inflow point, where native Se, (Ca,Sr)CO_3_, and radiobarite precipitate (Fig. 5). Also in the clay system, a decrease in *K*_d_ value occurs in the area affected by the deep groundwater, for those elements that are retained either by sorption onto illite and/or incorporation into solid phases (Sr, Cs, U, Ni, Th, C, and Ra, see Fig. 6).

The computed *K*_d_ values are not equal in the till and clay domains for most elements. In general, they are up to one order of magnitude higher in the clay domain for Sr and Cs and up to two orders of magnitude higher for Ni and Th. The stronger retention of these elements in the clay domain is explained by the larger amount of illite, and therefore of available sorption sites. In contrast, the *K*_d_ value of Ra is up to two orders of magnitude higher in the till domain due to radiobarite precipitation. Uranium and radiocarbon show *K*_d_ values of the same order of magnitude in both systems. In the till system, the most strongly retained radionuclides are Se and Cs, followed by Th and Ni, whereas U, Sr, C, and Ra are much less well retained (Fig. 5). In the clay system, Cs, Ni, and Th are strongly retained, whereas Sr, U, C, and Ra are less well retained (Fig. 6).

With the exception of Se, the computed *K*_d_ values in the till system are one or several orders of magnitude lower than the measured *K*_d_ in Forsmark till soils (see Sheppard et al. 2011 and Sohlenius et al. 2013 for methodology and results on site-specific *K*_d_). Computed *K*_d_ values of Ni and Cs in the clay system are in the range of those reported by Engdahl et al. (2008) for Forsmark lake sediments, whereas those for Th and Sr are one order of magnitude lower and those for U two orders of magnitude lower. However, measured *K*_d_ values in soils are not directly comparable with those of the modeled domains, due to differences in a number of environmental conditions, methods of extraction and parameters that could influence retention.

## Conclusions

Conceptual description and numerical simulations of radionuclide reactive transport in Forsmark till and clay deposits show that cation exchange and surface complexation on illite are active processes for the retention of several repository-derived radionuclides (U, Th, Ni, Cs, Sr). Furthermore, surface complexation on iron hydroxide is an active process able to retain U and Ni in the till. Another retention process is the incorporation of elements into mineral phases, such as C and Sr into a carbonate solid solution in both till and clay deposits, as well as the precipitation of uraninite in the clay and the precipitation of native selenium and radiobarite in the till. In the numerical models, Th, Ni, and Cs are the most retarded radionuclides, mainly through sorption onto illite, followed by U, C, Sr, and Ra. Selenium is strongly retained in the till domain and behaves conservatively in the clay domain. Niobium and Tc behave conservatively in both domains.

*K*_d_ values estimated from in situ measurements are usually taken as constant for a given site and sediment type, but computed results clearly show that *K*_d_ values are heterogeneous in space. The effective *K*_d_ values are determined directly from the modeled breakthrough curves at the discharge area of the model, so that they represent ‘upscaled’ *K*_d_ values quantifying retention in the whole domain between the source and the discharge area. Thus, calculation of effective *K*_d_ values in reactive transport simulations of this type constitutes an appropriate way to obtain parameter values for use in simplified box models, commonly used for performance assessment and dose calculations.

Finally, even though measured *K*_d_ values in Forsmark soils are not directly comparable with those of the modeled domains, the quantitative differences between measurements and model results indicate a need for further development of modeling capabilities, primarily concerning the ability to quantitatively represent processes that were not possible to consider in the present work.
